# Experience of Pediatricians and Pediatric Surgeons With Virtual Care During the COVID-19 Pandemic: Descriptive Study

**DOI:** 10.2196/34115

**Published:** 2022-06-15

**Authors:** Emma McCrady, Julie E Strychowsky, Jessica P Woolfson

**Affiliations:** 1 Schulich School of Medicine & Dentistry Western University London, ON Canada; 2 Department of Pediatrics London Health Sciences Centre Children's Hospital London, ON Canada; 3 Department of Otolaryngology-Head and Neck Surgery Western University London, ON Canada; 4 Division of Pediatric Gastroenterology London Health Sciences Centre Children's Hospital London, ON Canada

**Keywords:** virtual care, web-based care, COVID-19, pediatrics, pandemic, physicians, digital health, pediatricians, telehealth

## Abstract

**Background:**

Prior to the COVID-19 pandemic, in-clinic visits were the standard of care for pediatric physicians and surgeons at our center. At the pandemic onset, web-based care was adopted at an unprecedented scale and pace.

**Objective:**

This descriptive study explores the web-based care experience of pediatric physicians and surgeons during the pandemic by determining factors that supported and challenged web-based care adoption.

**Methods:**

This study took place at the Children’s Hospital at London Health Sciences Centre, a children’s hospital in London, Ontario, Canada, which provides pediatric care for patients from the London metropolitan area and the rest of Southwestern Ontario. The Donabedian model was used to structure a web-based survey evaluating web-based care experience, which was distributed to 121 department-affiliated pediatric physicians (including generalists and subspecialists in surgery and medicine). Recruitment occurred via department listserv email. Qualitative data were collected through discrete and free-text survey responses.

**Results:**

Survey response rate was 52.1% (63/121). Before the pandemic, few physicians within the Department of Paediatrics used web-based care, and physicians saw <10% of patients digitally. During March-May 2020, the majority transitioned to web-based care, seeing >50% of patients digitally. Web-based care use in our sample fell from June to September 2020, with the majority seeing <50% of patients digitally. Telephone and Ontario Telemedicine Network were the platforms most used from March to September 2020. Web-based care was rated to be convenient for most providers and their patients, despite the presence of technical difficulties. Challenges included lack of physical exam, lower patient volumes, and poor patient digital care etiquette. Regardless of demographics, 96.4% (116/121) would continue web-based care, ideally for patients who live far away and for follow-ups or established diagnoses.

**Conclusions:**

Transition to web-based care during COVID-19 was associated with challenges but also positive experiences. Willingness among pediatricians and pediatric surgeons to continue web-based care was high. Web-based care experiences at our center could be improved with patient education and targeting select populations. Future research is needed to improve practice efficiency and to inform regulatory guidelines for web-based care.

## Introduction

Web-based care has been defined as any interaction between patients or members of their circle of care, occurring remotely, using any forms of communication or information technologies, with the aim of facilitating or maximizing the quality and effectiveness of patient care [1]. Though Canadian physicians have been using technologies for delivery of web-based care as early as the 1970’s [2], prior to the COVID-19 pandemic, there remained significant barriers to widespread delivery of web-based care across the country [3]. Although web-based care has demonstrated utility in several pediatric subspecialties [4], before March 2020, the standard of care for pediatricians affiliated with our center was in-clinic visits, with web-based visits limited to the Ontario Telemedicine Network for select patients in remote locations. Additionally, given the sparse uptake of web-based care across pediatric subspecialties at our center, little was known about local web-based care practice patterns before the pandemic.

With the onset of the COVID-19 pandemic, web-based care was adopted at an unprecedented scale and pace to mitigate and manage the risk of spread of the disease [5]. Regardless of previous experience with web-based care, physicians of nearly all specialties and disciplines were required to adopt some proportion of digital practice as a means of maintaining a continuum of patient care [6]. Despite initial positive feedback, the sudden rush to web-based systems carried the risk of diminishing quality of clinical care [7]. At the time of our study, little was known of the impact of this shift for providers, particularly pediatricians. Therefore, this descriptive study sought to qualify the web-based care experiences of local pediatricians during the early stages of the COVID-19 pandemic (March to September 2020), with the intention of implementing web-based care clinical practice changes at the department level.

## Methods

### Study Design

The Donabedian model for health care quality improvement was used to guide questions within the survey on the Qualtrics platform (Multimedia Appendix 1). This survey was then used to evaluate the web-based care experience of staff pediatric physicians and surgeons through their responses. In this survey, web-based care was defined as any interaction between patients or members of their circle of care, occurring remotely, using any forms of communication or information technologies [1]. Within the Donabedian model, “structure” refers to assessment of health care settings, qualifications of providers, and administrative systems through which care is provided. In our survey, this was assessed with demographic questions, web-based care settings, start-up costs, and platforms used. “Process” refers to the elements of care delivered within clinician-technical and clinician-interpersonal relationships. This was addressed in our survey with questions about percentage of care provided virtually, as well as free text responses related to patient care. Finally, “outcome” refers to achievement of goals of care, with indicators such as satisfaction, safety, and good use of resources. In our survey, our primary outcome was physician experience with web-based care, measured by positive experience with web-based care, negative experience with web-based care, and willingness to continue to provide web-based care in the future. Phrasing of survey questions was balanced to include positive and negative options to avoid leading questions, and the survey was pilot tested by a non-London Health Sciences Centre (LHSC)–affiliated pediatrician for readability and content.

### Ethics Approval

Research ethics exemption was granted by the Office of Human Research Ethics on behalf of Western University’s Research Ethics Board. As this study is a part of a larger quality improvement initiative, this was granted under the Quality Assurance/Quality Improvement/Program Evaluation classification.

### Study Setting

This study took place at LHSC, a children’s hospital in London, Ontario, Canada, which provides pediatric care for patients from the London metropolitan area and the rest of Southwestern Ontario. This survey was introduced to the Department of Paediatrics in September 2020 to assess web-based practice patterns from March 2020 to September 2020.

### Recruitment and Data Collection

Recruitment for the study occurred via email in September 2020 to all LHSC-affiliated pediatric physicians and surgeons within the department’s listserv email database. The Department of Paediatrics email listserv comprises 121 staff physicians, including generalists, subspecialists, pediatric surgeons, as well as academic and community physicians. A follow-up email was sent through the same listserv 2, 4, and 6 weeks after the initial email to prompt further response, with a deadline of 6 weeks total to complete the survey.

### Data Analysis

The web-based survey was analyzed through the QualitricsXM platform and Microsoft Excel (version 16.16.5). The results were collated, and descriptive statistics for numerical data were calculated. Partially completed responses to the survey were excluded from analysis, noted as “no response” in Tables 1-5. Free text responses were reviewed by the research team to identify themes and provide further support to numerical data (Multimedia Appendix 2).

## Results

### Survey Response

Of the 121 pediatric physicians, 63 (52.1%) responded. Demographic information of the respondents may be viewed in Table 1. The respondents were primarily subspecialists working at an academic institution. This is consistent with the demographics of the total listserv database, which comprises 19 (15.7%) community generalists and 102 (84.3%) academic subspecialists in both pediatric surgery and medicine.

**Table 1:** Demographic characteristics of the survey respondents.

Characteristics			Values, n (%)
**Primary practice location**			
	Academic Children’s Hospital	56 (88.9)	
	Community Hospital	0 (0)	
	Community Clinic	5 (7.9)	
	Other	2 (3.2)	
**Practice type**			
	General	10 (15.9)	
	Subspecialist	53 (84.1)	
**Years in practice**			
	≤5	17 (27.0)	
	6-10	9 (14.3)	
	11-20	20 (31.7)	
	≥21	17 (27.0)	

### Adoption of Web-Based Care During the COVID-19 Pandemic

While the majority of respondents had no prior web-based care experience (Table 2) during the first wave of the pandemic (March-May 2020), almost all respondents transitioned to web-based care, with the majority seeing more than half of their patients digitally. By summer (May-September 2020) web-based care use declined, with the majority of physicians seeing less than 25% of their patients digitally. Reported percentages of web-based care are based on physician estimates.

Respondents were more likely to use synchronous web-based care methods with live audio or video feedback over asynchronous methods such as email or secure messaging software (Table 3). The most commonly used web-based care platforms in our sample were telephone and the Ontario Telemedicine Network, which were also selected as the most popular platforms ideally used in the future.

**Table 2:** Web-based care practice patterns.

Characteristics		Values, n (%)
**Prior** **web-based** **care experience**		
	Yes	19 (30.2)
	No	44 (69.8)
**Adopted** **web-based** **care during pandemic**		
	Yes	59 (93.7)
	No	4 (6.3)
**Percentage of practice via** **web-based** **care (March-May 2020)**		
	Closed practice	1 (1.6)
	0%	4 (6.3)
	1%-10%	8 (12.7)
	11%-25%	6 (9.5)
	26%-50%	3 (4.8)
	51%-75%	6 (9.5)
	75%-100%	28 (44.4)
	No response	6 (9.5)
**Percentage of practice via** **web-based** **care (June-September 2020)**		
	Closed practice	0 (0)
	0%	2 (3.2)
	1%-10%	15 (23.8)
	11%-25%	15 (23.8)
	26%-50%	8 (12.7)
	51%-75%	12 (19.0)
	75%-100%	4 (6.3)
	No response	6 (9.5)

**Table 3:** Physician platform use.

Characteristics				Values, n (%)
**Platforms used to provide care (March-September 2020)**				
	**Synchronous**			
		Doxy	4 (6.3)	
		Cisco Webex	17 (27.0)	
		Zoom	16 (25.4)	
		OTN^a^	35 (55.6)	
		Facetime	7 (11.1)	
		Telephone	41 (65.1)	
		Microsoft Teams	1 (1.0)	
	**Asynchronous**			
		Secure messaging	3 (4.8)	
		Email	18 (28.6)	
		No response	10 (15.9)	
**Anticipated platform use (September 2020 onward)**				
	**Synchronous**			
		Doxy	4 (7.4)	
		Cisco Webex	12 (22.2)	
		Zoom	10 (18.5)	
		OTN	39 (72.2)	
		Facetime	1 (1.9)	
		Telephone	34 (63.0)	
		Microsoft Teams	2 (1.0)	
	**Asynchronous**			
		Secure messaging	3 (3.7)	
		Email	18 (33.3)	
		No response	9 (14.3)	

^a^OTN: Ontario Telemedicine Network.

### Challenges of Web-Based Care Use

Free text responses by survey respondents provided insight into challenges during their adoption of web-based care from March 2020 onward (Multimedia Appendix 2). Those who felt web-based care did not work well for their patients frequently cited the inability to perform a physical examination and the associated diagnostic uncertainty as challenges. The respondents reported disappointment with a lack of respect of the web-based encounter, citing examples of patients answering digital calls in shopping malls or poolside. The majority of physicians did not feel they could see a higher volume of patients digitally (Table 4). Technical difficulties and lack of adequate compensation were additional challenges.

**Figure 1:**
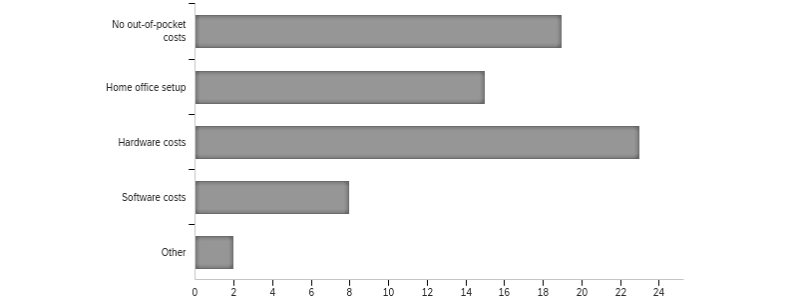
Costs associated with the transition to web-based care.

With the transition to web-based care, out-of-pocket costs were encountered by 64% (34/63) of the respondents (Figure 1). While the exact cost associated was not quantified in our study, these tended to be one-time start-up costs (ie, web camera) versus recurring fees.

**Table 4:** Provider opinions on web-based care, March 2020 to September 2020.

Question	Strongly agree, n (%)	Somewhat agree, n (%)	Neither agree nor disagree, n (%)	Somewhat disagree, n (%)	Strongly disagree, n (%)	No response, n (%)
Virtual care does not work well for my patient population.	4 (7.7)	16 (30.8)	11 (21.2)	12 (23.1)	9 (17.3)	11 (17.4)
Technical difficulties are a challenge for me in my virtual practice.	3 (5.8)	19 (36.5)	14 (26.9)	14 (26.9)	2 (3.8)	11 (17.4)
I am compensated adequately for the virtual care I provide.	7 (13.5)	12 (23.1)	14 (26.9)	7 (13.5)	12 (23.1)	11 (17.4)
I see a higher volume of patients virtually.	4 (7.7)	6 (11.5)	10 (19.2)	15 (28.8)	17 (32.7)	11 (17.4)
I find it difficult to use virtual care platforms.	1 (1.9)	9 (17.0)	13 (24.5)	13 (24.5)	17 (32.1)	10 (15.9)
Virtual care is convenient for me.	13 (24.5)	26 (49.1)	5 (9.4)	6 (11.3)	17 (5.7)	10 (15.9)
Patients find it difficult to use virtual care platforms.	4 (7.7)	15 (28.8)	15 (28.8)	15 (28.8)	3 (5.8)	11 (17.5)
My patients are safer as a result of virtual care.	8 (15.4)	16 (30.8)	20 (38.5)	6 (11.5)	2 (3.8)	11 (17.5)
Technical difficulties are a challenge for my patients.	9 (17.3)	23 (44.2)	11 (21.2)	7 (13.5)	2 (3.8)	11 (17.5)
Patients are satisfied with the transition to virtual care.	12 (23.1)	32 (61.5)	6 (11.5)	2 (3.8)	0 (0)	11 (17.5)
Patients are compliant with virtual care visits.	4 (7.7)	31 (59.6)	10 (19.2)	7 (13.5)	0 (0)	11 (17.5)
Virtual care is convenient for my patients.	19 (36.5)	30 (57.7)	3 (5.8)	0 (0)	0 (0)	11 (17.5)

### Facilitators of Web-Based Care Use

Web-based care was rated as convenient for both physicians and patients, despite the presence of technical difficulties (Table 4). Based on free text feedback (Multimedia Appendix 2), the physicians who viewed web-based care as appropriate for their patient populations were typically those with a primary focus on history and less reliance on a physical exam.

### Future Use

Regardless of practice location, specialty, years in practice, or prior experience with web-based care, 96.4% (n=54) of the survey respondents would continue to provide web-based care in the future (Table 5). The majority of respondents would ideally see less than half of their patients digitally moving forward and identified patients who live far away and follow-ups or established diagnoses as ideal populations to serve digitally. Patient satisfaction, improved technology, and provincial or national policies are top motivating factors for continued web-based care use.

**Table 5:** Provider opinions on web-based care use from September 2020 onward.

Characteristics			Values, n (%)
**Plans to continue to provide** **web-based** **care**			
	Yes	54 (96.4)	
	No	2 (3.6)	
	No response	7 (11.1)	
**Ideal percentage of future** **web-based** **patient interactions**			
	0%	3 (5.4)	
	1%-10%	17 (30.4)	
	11%-25%	20 (35.7)	
	26%-50%	12 (21.4)	
	51%-75%	3 (5.4)	
	75%-100%	1 (1.8)	
	No response	7 (11.1)	
**Patients best served virtually**			
	Nonacute	21 (51.2)	
	New consults	9 (22.0)	
	Follow-ups or established diagnosis	30 (73.2)	
	Patients who live far away	37 (90.2)	
	Immunocompromised patients	21 (51.2)	
	Patient preference	22 (53.7)	
	No response	22 (34.9)	
**Motivating factors for continued** **web-based** **care integration**			
	Department-wide policy	23 (42.6)	
	Financial incentive	29 (53.7)	
	Patient satisfaction	36 (66.7)	
	Better technology	33 (61.1)	
	Other (please specify)	9 (16.7)	
	Provincial or national policy	32 (59.3)	
	LHSC^a^ endorsement of a specific platform	14 (25.9)	
	Training sessions for how to optimize web-based care	11 (20.4)	
	Ability to incorporate trainees into web-based care	28 (51.9)	
	No response	9 (14.3)	

^a^LHSC: London Health Sciences Centre.

## Discussion

### Principal Results

Our study provides a unique insight into how pediatric physicians and surgeons at our center adjusted to a sudden virtualization of health care and their attitudes toward integration of web-based clinical practice in the future. With the onset of the COVID-19 pandemic, web-based care was adopted swiftly, exemplified by its almost-unanimous adoption by pediatric physicians and surgeons at our center, over half of whom provided the majority of their care digitally. Interestingly, months later, in the summer of 2020, practice patterns changed to reduce the percentage of web-based visits, with over half of physicians and surgeons seeing less than 50% of their patients virtually. Those who were the most enthusiastic about web-based care were academic subspecialists who spend a greater proportion of their visits taking history, see patients with less acuity, and do not rely heavily on a physical examination. Looking to the future, buy-in for web-based care was high, with almost all survey respondents willing to continue to provide web-based care in the future, regardless of demographic factors and prior experience with web-based care.

### Comparison With Prior Work

Our results contribute to the existing literature demonstrating increased prevalence of web-based care visits during the COVID-19 pandemic, through examination of patterns of pediatric physician and surgeon provision. As evidenced by Bhatia and colleagues, prior to the pandemic, the delivery of care through web-based means was limited, with only a small number of Ontario physicians offering visits. However, during the first 6 months of 2020, the majority of Ontario residents had used web-based care for at least one appointment [8]. Furthermore, those who had connected with their doctor digitally during that time reported a 91% satisfaction rate [9]. Similar to provincial patterns, within our cohort, telephone visits were the primary method of contact during the first wave of the pandemic [8]. Interestingly, previous studies reporting on physician preference report that although audio or video visits represent the closest substitute to in-person visits, they are less convenient for providers when compared to asynchronous messaging, which allows time to review patient cases and respond when available [10,11]. In our study, asynchronous messaging represented a smaller proportion of web-based care visits, perhaps due to lack of familiarity, lack of department endorsement of these platforms, and perhaps poorer financial incentives. However, asynchronous delivery of care does offer the potential to reduce issues identified by our respondents, such as reduced web-based care patient volumes and poor patient etiquette.

Throughout the literature, it has been determined that web-based care works well in populations where a significant portion of the visit is spent taking history, with less reliance on a physical exam, which was supported by free text responses from our sample [12]. The challenges identified in our study, including lack of physical examination and poor patient etiquette, were supported by other studies of web-based care during the pandemic [4,13]. In addition to factors challenging physician adoption of web-based care, certain patient-level factors were identified as prohibitive to widespread use of web-based care. At our center specifically, which sees a high volume of Amish and Mennonite populations, accessibility in terms of equipment availability, technological literacy, and patient engagement are concerns. These barriers may be experienced by additional patient groups, such as newcomers, patients experiencing poverty and homelessness, and those with physical or intellectual disabilities.

Issues with web-based care etiquette were not unique to our center, as evidenced by a recent article outlining the experiences of physicians across the country renegotiating the rules of engagement in their web-based practices [14]. Physicians felt that, without better patient education, conducting a detailed interview, passive evaluation, or facilitated evaluation necessary to create a thorough assessment and treatment plan would be extremely difficult. While there is a wealth of patient information tools for web-based care readiness available, we found that standardization of pediatric-specific, web-based care patient education tools was lacking in our center. Furthermore, while physician education on web-based care has been well documented in the literature [6,15,16], further research is needed to explore the impact of patient education. Based on survey responses and lack of available data on the topic, our department plans to create a pediatric-specific web-based etiquette tool, with the goal of measuring the impact of web-based care on physician experience.

### Limitations

Our study is limited by our response rate, with only 52.1% (63/121) of pediatric physicians and surgeons at our center having completed the survey. Though our convenience sample was representative of all durations of practice and included those with and without previous experience in web-based care, sampling method and response rate limit the generalizability of data across the Department of Paediatrics as a whole, and selection bias may have occurred. Given the small number of participants, we were unable to provide any subanalysis. Furthermore, only a small number of community pediatricians (7.9%) offered responses, with no response provided by those working in community hospitals. Further research is needed to explore the needs of community pediatricians and how the provision of web-based care may differ between academic and community practices. It should be noted that this study is only representative of a short period of time, March 2020 to September 2020. Additionally, during this period, we were unable to validate true percentages of web-based care used, and recall bias may have influenced responses to these questions. As COVID-19 continues to challenge the provision of in-person care, further research is needed to better understand current practice patterns and how they have changed over the last 2 years to improve practice efficiency and to inform regulatory guidelines for web-based care.

### Conclusion

In conclusion, the transition to web-based care during the early COVID-19 pandemic period at our center was associated with challenges, but also with positive experiences. Willingness to continue web-based care among pediatric physicians and surgeons is high. It was determined that select populations, particularly follow-ups and established diagnoses, may benefit more from web-based care compared with other groups such as new consults and higher acuity cases. Pediatric physicians’ web-based care experiences at our center could be improved with greater patient education, improved technology, and provincial or national policies to guide web-based practice. Future directions include the development of a web-based care patient education tool to improve patient and provider experience.

## Appendix

Multimedia Appendix 1 Survey questions.

Multimedia Appendix 2 Themes and illustrative quotes.

## Abbreviations

LHSC: London Health Sciences Centre
