# Supplementary material for: Experience of Pediatricians and Pediatric Surgeons With Virtual Care During the COVID-19 Pandemic: Descriptive Study
Source: JMIR Pediatr Parent. 2022 Jun 15;5(2):e34115. doi: 10.2196/34115 (PMC9202653; doi:10.2196/34115)
Supplement: Multimedia Appendix 1 [file pediatrics_v5i2e34115_app1.docx]

**MULTIMEDIA APPENDIX I: Survey Questions**

**Virtual Care: A Quality Improvement Project on the Experience of Paediatricians during the COVID Pandemic**

You are invited to take part in a quality improvement initiative to help us better understand your experience as a virtual care provider. Your participation and responses to this survey will provide us with valuable information which may guide changes to virtual clinical practice within the department of paediatrics as we move further away from the COVID-19 pandemic.

Would you like to continue?*

- Yes
- No

**Part 1: Demographics**

What is your primary practice location?

- Academic Children's Hospital
- Community Hospital
- Community Clinic
- Other (please specify) ________________________________________________

Which of the following best describes your practice?

- General
- Subspecialist

How many years have you been in practice?

- ≤5
- 6-10
- 11-20
- ≥21

**Part 2: Pre-COVID**

Prior to the COVID-19 Pandemic, was virtual care a part of your practice?

- Yes
- No

If yes: What percentage of patient interactions were conducted virtually?

- 0%
- 1-10%
- 11-25%
- 26-50%
- 51-75%
- 76-100%

**Part 3: During COVID**

During the COVID-19 Pandemic, did you use virtual care as a part of your practice?

- Yes
- No

If yes: Which platforms did you use to provide care? Please check all that apply.

- Doxy
- Cisco Webex
- Zoom
- OTN
- Facetime
- Skype
- Telephone
- Secure messaging
- Email
- Other (Please specify) ________________________________________________

If yes: What was your virtual care setting? Please check all that apply.

- Home/home office
- Office at work location
- Other space at work location (i.e. designated room)
- Other (Please specify)

If yes: Did you incur any out of pocket costs associated with the transition to virtual care? Please check all that apply.

- No out of pocket costs
- Home office setup (paper, pens, lighting, seating, desk space etc)
- Hardware costs (computer, video camera, microphone, speakers, earphones, etc.)
- Software costs (secure messaging service, video conferencing service, etc.)
- Other (Please specify) ________________________________________________

If yes: Please respond to the following statements regarding **your virtual care environment** during COVID:

|  | Strongly agree | Somewhat agree | Neither agree nor disagree | Somewhat disagree | Strongly disagree |
| --- | --- | --- | --- | --- | --- |
| I work in an environment with adequate lighting for videoconferencing |  |  |  |  |  |
| I have challenges with internet connection when conducting virtual care appointments |  |  |  |  |  |
| I am pleased with the sound quality of my virtual care platform |  |  |  |  |  |
| Patients are pleased with the sound quality of my virtual care platform |  |  |  |  |  |
| Equipment availability (camera, microphone) is a challenge for my patients |  |  |  |  |  |
| Equipment availability (camera, microphone) is a challenge for me in my virtual practice |  |  |  |  |  |
| I have concerns about privacy related to virtual care |  |  |  |  |  |
| Patients are concerned about their privacy related to virtual care |  |  |  |  |  |

During the height of COVID-19 pandemic in March-May 2020, what percentage of your practice WAS provided via virtual care?

- I closed my practice during this time
- 0%
- 1-10%
- 11-25%
- 26-50%
- 51-75%
- 76-100%

Currently in the COVID-19 pandemic (June-September 2020) what percentage of your practice is provided via virtual care?

- My practice is closed
- 0%
- 1-10%
- 11-25%
- 26-50%
- 51-75%
- 76-100%

Please respond to the following statements regarding **your patients' experience** with virtual care during COVID:

|  | Strongly agree | Somewhat agree | Neither agree nor disagree | Somewhat disagree | Strongly disagree |
| --- | --- | --- | --- | --- | --- |
| Patients are satisfied with the transition to virtual care |  |  |  |  |  |
| Patients are compliant with virtual care visits |  |  |  |  |  |
| Patients find it difficult to use virtual care platforms |  |  |  |  |  |
| My patients are safer as a result of virtual care |  |  |  |  |  |
| Technical difficulties are a challenge for my patients |  |  |  |  |  |
| Virtual care is convenient for my patients |  |  |  |  |  |

Please respond to the following statements regarding **your experience** providing virtual care during COVID:

|  | Strongly agree | Somewhat agree | Neither agree nor disagree | Somewhat disagree | Strongly disagree |
| --- | --- | --- | --- | --- | --- |
| I am satisfied with the transition to virtual care |  |  |  |  |  |
| Virtual care is convenient for me |  |  |  |  |  |
| I find it difficult to use virtual care platforms |  |  |  |  |  |
| I see a higher volume of patients virtually |  |  |  |  |  |
| I have received adequate training to provide care virtually |  |  |  |  |  |
| I feel safer at work as a result of the transition to virtual care |  |  |  |  |  |
| I am compensated adequately for the virtual care I provide |  |  |  |  |  |
| I have concerns about liability related to virtual care |  |  |  |  |  |
| Virtual care does not work well for my patient population |  |  |  |  |  |

**Part 4: Expectations Of Virtual Care In Future Practice**

In the future following the COVID-19 pandemic, will you continue to provide virtual care?

- Yes
- No

If yes: Which platforms do you plan to use to provide virtual care? Please check all that apply.

1. Doxy
2. Cisco Webex
3. Zoom
4. OTN
5. Facetime
6. Skype
7. Telephone
8. Secure messaging
9. Email
10. Other (Please specify) ________________________________________________

Ideally, what percentage of patient interactions do you envision remaining virtual?

- 0%
- 1-10%
- 11-25%
- 26-50%
- 51-75%
- 76-100%

If yes: What is your preferred practice setting for ongoing virtual care post-pandemic

- Home/home office
- Office at work location
- Other space at work location (i.e. designated room)
- Other (Please specify) ______________________________________________

How will you modify your practice post-COVID?

- Continue to offer virtual care to all patients
- Continue to offer virtual care to select patients
- Return to practice as it was pre-COVID

*If “Continue to offer virtual care to select patients” is chosen*: Which patients would you continue to care for virtually?

- Non-acute
- New consults
- Follow-ups/established diagnosis
- Patients who live far away
- Patients with increased risk of infection/COVID
- Patient preference

What would make you more inclined to continue to use virtual care in your practice on an ongoing basis? Please check all that apply.

- Provincial or national policy
- Department-wide policy
- Financial incentive
- Patient satisfaction
- Better technology
- LHSC endorsement of a specific platform
- Training sessions for how to optimize virtual care
- Ability to incorporate trainees into virtual care
- Other (please specify):

*If “Use of a specific platform” is chosen*: Do you have a suggested platform for widespread future use:

- Doxy
- Cisco Webex
- Zoom
- OTN
- Facetime
- Skype
- Telephone
- Secure messaging
- Email
- Other (Please specify) __________________

Please feel free to provide any additional comments regarding your thoughts on paediatric virtual care

________________________________________________

We thank you for your time spent taking this survey. Your response has been recorded.
