# Supplementary material for: Experience of Pediatricians and Pediatric Surgeons With Virtual Care During the COVID-19 Pandemic: Descriptive Study
Source: JMIR Pediatr Parent. 2022 Jun 15;5(2):e34115. doi: 10.2196/34115 (PMC9202653; doi:10.2196/34115)
Supplement: Multimedia Appendix 2 [file pediatrics_v5i2e34115_app2.docx]

**MULTIMEDIA APPENDIX II: Themes and illustrative quotes**

| *Theme & Subtheme* | *Quotes* |
| --- | --- |
| Challenges with Virtual Care Use: Diagnostic Uncertainty | “The inability of a physical examination forms a major limitation. Based on my own research, an in person visit needs to occur after two virtual care visits. Children do not appreciate the virtual care quite as much as adults.” *- Subspecialist, >21y in practice, pre-covid virtual care experience.*  “Most of my patients require in person testing or physical exam that cannot be done virtually. Virtual visits could be appropriate for 10-20% of patients but otherwise they just serve to defer visits, not replace them. They are convenient for out of town patients.” *- Subspecialist, 6-10y in practice, no pre-covid virtual care experience.*  “Although virtual care has been a good Bandaid solution during COVID, virtual care cannot replace an in person visit for most conditions. Patients really need to be examined, and you get a very different sense of well/unwell especially in paediatrics (where often on OTN or telephone the child is absent or only briefly present). While I may continue to do some care virtually, all patients in my practice will at some point need to be seen in person. I see virtual as be complementary for certain scenarios, not a replacement for any scenario in its entirety (and I feel very strongly that the decision to have a patient or any percentage of care delivered virtually needs to be up to the individual physician - NOT dictated by an expectation for a percentage of virtual care by leadership.”*- Subspecialist, <5y in practice, no pre-covid virtual care experience*  “Virtual care does not work well for other forms of work, such as walk-in visits in which there is a much higher risk of missing something important due to lack of physical exam.”*- Generalist, <5y in practice, no pre-covid virtual care experience* |
| Challenges with Virtual Care Use: Patient Virtual Etiquette | “If virtual care will continue I think some patient education about it being a viable and legitimate way to provide care is important. I have had several no shows and patients not always treating it like a true appointment.”*- Subspecialist, <5y in practice, no pre-covid virtual care experience*  “We should teach patients the importance and respect virtual consultations. They cannot be allowed to take calls in a shopping mall for example. It devalues the sanctity, professionalism and mutual respect of in person consultations.”*- Subspecialist, 11-20y in practice, yes pre-covid virtual care (1-10%)* |
| Challenges with Virtual Care Use: Logistical Issues | “Logistically challenging to mix in-person and virtual care. You are inevitably running late for phone calls at the end of the day. Or else, you have to set aside additional dates/times outside of your usual clinic for virtual visits which takes away from other responsibilities.” *- Subspecialist,>5y in practice, no pre-covid virtual care experience*  “The departments need to provide more financial incentives and at the very minimum more IT support for virtual subspecialty care for paediatric patients. As physicians, we also need more legal counsel and more safeguards for potential medicolegal issues as it relates to virtual care. Currently, this is lacking both from the CMPA and from our departments. These pitfalls are heightened 10 fold for community practictioners.”*- Subspecialist, 11-20y in practice, no pre-covid virtual care experience* |
| Facilitators of Virtual Care Use: Patient/Physician Convenience | “ Paediatric virtual care works well for our patients. The parent feedback has been overwhelmingly positive. Having Cisco WexEx linked through the hospital so the patient can be “registered” is extremely valuable for tracking and charting. Most patients do well setting up the meeting. Those who find the technology challenging can receive a phone call.”  *- Subspecialist, 11-20y in practice, no pre-covid virtual care experience*  “In my specialty it has worked out really well since we usually spend a significant amount of time taking the history. In the outpatient clinic, this would often result in the child acting up, becoming restless, exploring places or trying to escape from the room. With OTN, the parent can have someone else watching the child in the room, where toys, etc. are easily accessible. Much less distracting for parent or upsetting for child. Then if we need to examine the child, it is a quick outpatient visit and arrange for bloodwork. Trainees can sit in on the appointment or take the history themselves.” *- Subspecialist, >21y in practice, no pre-covid virtual care experience*  “Virtual care works well for a subset of my patients (developmental paeds consults)” - *Generalist, <5y in practice, no pre-covid virtual care experience* |
